# Structural displacement model of chitooligosaccharide transport through chitoporin

**DOI:** 10.1016/j.jbc.2023.105000

**Published:** 2023-07-01

**Authors:** Surapoj Sanram, Anuwat Aunkham, Robert Robinson, Wipa Suginta

**Affiliations:** School of Biomolecular Science and Engineering (BSE), Vidyasirimedhi Institute of Science and Technology (VISTEC), Rayong, Thailand

**Keywords:** chitin, chitoporin, sugar-specific channel, *Vibrio* spp

## Abstract

*Vh*ChiP is a chitooligosaccharide-specific porin identified in the outer membrane of *Vibrio campbellii* type strain American Type Culture Collection BAA 1116. *Vh*ChiP contains three identical subunits, and in each subunit, the 19-amino acid *N*-terminal segment serves as a molecular plug (the “*N*-plug”) that controls the closed/open dynamics of the neighboring pores. In this study, the crystal structures of *Vh*ChiP lacking the *N-*plug were determined in the absence and presence of chitohexaose. Binding studies of sugar–ligand interactions by single-channel recordings and isothermal microcalorimetry experiments suggested that the deletion of the *N*-plug peptide significantly weakened the sugar-binding affinity due to the loss of hydrogen bonds around the central affinity sites. Steered molecular dynamic simulations revealed that the movement of the sugar chain along the sugar passage triggered the ejection of the *N*-plug, while the H-bonds transiently formed between the reducing end GlcNAc units of the sugar chain with the *N*-plug peptide may help to facilitate sugar translocation. The findings enable us to propose the structural displacement model, which enables us to understand the molecular basis of chitooligosaccharide uptake by marine *Vibrio* bacteria.

*Vibrio campbellii* (formerly known as *Vibrio harveyi*) is a noncholera, bioluminescent, virulent pathogen that causes a lethal disease, known as luminous *Vibriosis*, in both wild and cultured aquatic animals (1). *V. campbellii* is a fast-growing bacterium, which plays a significant role in the recycling of chitin and in this way, contributes to the carbon and nitrogen balance between the oceans and the earth. Like other marine *Vibrio* spp. (2, 3, 4), *V. campbellii* possesses an active chitin catabolic pathway, which allows the bacterium to utilize chitin as its sole carbon source. The chitin catabolic cascade of *V. campbellii* contains highly effective chitin-degrading enzymes (5, 6), and the degradation products are subsequently internalized by the cells. Chitin is first degraded to chitooligosaccharides ((GlcNAc)_n_, with n ≥2) and GlcNAc by chitinases (7, 8). Small sugar molecules, such as *D*-GlcNAc and (GlcNAc)_2_, are thought to pass through the outer membrane (OM) of the bacterial cells through protein channels that serve as general diffusion porins (9), while larger chitooligosaccharides ((GlcNAc)_3,4,5,6_) are taken up through a chitooligosaccharide-specific channel, known as chitoporin or ChiP (10, 11). The transported chitooligosaccharides are further degraded by chitin dextrinase (12) or exo-β-N-acetylglucosaminidases (6, 13), generating the final hydrolytic products *D*-GlcNAc and (GlcNAc)_2_, which are further translocated by a (GlcNAc)-specific phosphotransferase system transporter and a (GlcNAc)_2_ ABC transporter (14, 15, 16), respectively. The chitin catabolic cascade of marine *Vibrio* sp. is known to be operated by (GlcNAc)_2_-inducible genes that are tightly controlled by a two-component membrane-bound histidine kinase, identified as the chitin sensor (9). We previously identified and functionally characterized the first OM ChiP (*Vh*ChiP) from *V. campbellii* type strain American Type Culture Collection BAA-1116 (11). *Vh*ChiP is a sugar-specific OM porin with the ability to selectively transport a range of chitooligosaccharides, chitohexaose being the most preferred (11, 17). The average single-channel conductance of *Vh*ChiP was 1.8 ± 0.3 nS in 1 M KCl electrolyte, and each channel contains multiple GlcNAc-binding sites within the pore (18, 19). We previously determined the X-ray crystal structures of the OM-expressed and *in vitro*–refolded *Vh*ChiP in the absence and presence of chitotetraose and chitohexaose (PDB IDs: 5MDO, 5MDP, 5MDQ, 5MDR, 5MDS) (20). *Vh*ChiP consists of three identical β-barrels, each containing 16 β-strands connected on the extracellular side by eight hydrophilic loops and on the periplasmic side by eight short hydrophobic turns. In the structure of *Vh*ChiP complexed with chitohexaose, the sugar chain was found to extend throughout the extracellular side toward the periplasmic side and to interact exclusively with the multiple affinity sites. The most striking observation from our previous crystallographic data was that the *N* termini of the trimeric channel contained short helices of nine amino acids (Asp^1^–Lys^9^) that served as molecular plugs (the so-called “*N*-plugs”). In the OM-expressed channel (PDB ID: 5MDQ), all three subunits were plugged in such a manner that the *N*-plug of one subunit occluded the periplasmic half of the neighboring pore. However, in the open (*in vitro*–folded) channel (PDB ID: 5MDO), all three *N*-plugs were ejected from the trimeric pores.

The observation of the *N*-plug inside the OM-expressed *Vh*ChiP channel, but outside the sugar-bound channel, led us to hypothesize that entry of the sugar molecule into the protein pore may trigger the ejection of the *N*-plug. In this study, we performed structural determinations of the *N* terminally truncated *Vh*ChiP in the absence and presence of the preferred substrate (chitohexasose) and analyzed the protein–ligand interactions. We further employed time-resolved single-channel electrophysiology and isothermal microcalorimetry (ITC) to investigate sugar–channel interactions in the truncated channel and the Asp^1^ mutant and related the binding affinity to the structural data. In the last part of our study, we employed steered molecular dynamic (SMD) simulations to follow the movement of the sugar and its interactions with the *N*-plug inside the protein pore. Combining the data from protein crystallography, single-channel electrophysiology, and SMD simulations, we constructed a structural model for sugar translocation through the OM of marine *Vibrio* bacteria.

## Results

### The crystal structure of the *N*-terminal truncated *Vh*ChiP shows that chitohexaose is fully extended inside the channel lumen

**Figure 1:**
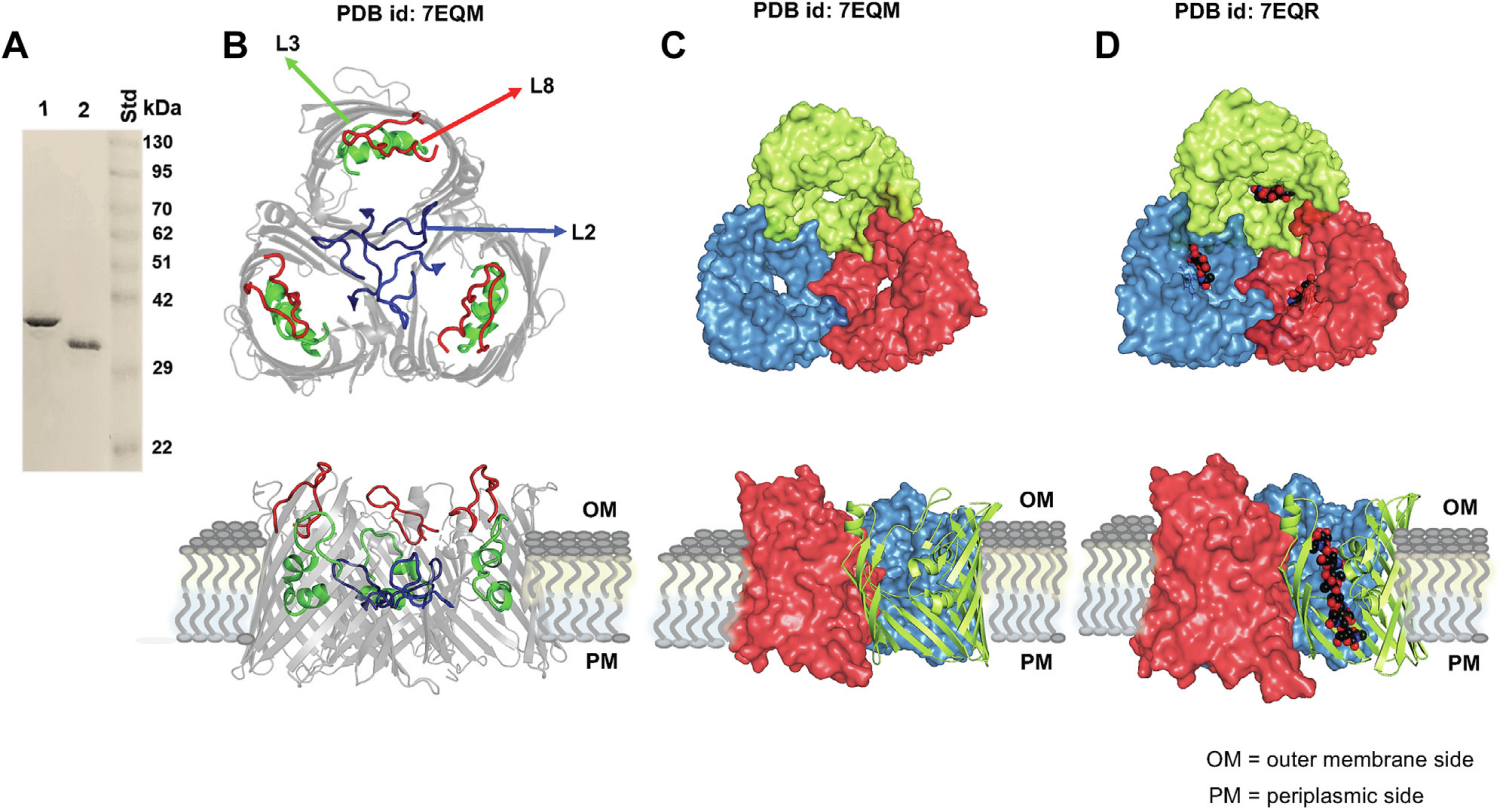
**Purification and structural determination of truncated *Vh*ChiP without and with sugar ligand.***A*, migration of the purified WT (*track 1*) and truncated *Vh*ChiP (*track 2*) relative to molecular weight standard proteins (track Std). *B*–*D*, X-ray crystal structures of the truncated *Vh*ChiPs. The *upper figures* are top views and *lower figures* are side views of the structures. *B*, cartoon representation of the ligand-free truncated *Vh*ChiP (PDB: 7EQM), showing the locations of three prominent loops: *red* for L8, *blue* for L2, and *green* for L3. *C*, surface representation of the ligand-free truncated structure (PDB: 7EQM. *D*, surface representation of the truncated structure in complex with chitohexaose (PDB: 7EQR). Chitohexaose is shown in a space-filling model, with atoms C represented in *black*, O in *red*, and N in *blue*. Each subunit is shown in a different color.

The truncated *Vh*ChiP channel, lacking nine *N*-terminal amino acid residues, was successfully expressed in the OM of the omp-deficient *Escherichia coli* BL21 (Omp8) Rosetta host. The OM-expressed *Vh*ChiP was extracted with 2% (*w/v*) SDS, followed by 3% (*v/v*) poly(ethylene glycol) octyl ether (octyl POE), then further purified to homogeneity by gel filtration. Figure 1*A* shows a representative SDS-PAGE gel, with a single Coomassie-stained band of WT *Vh*ChiP that migrated to below 42 kDa (Fig. 1*A*, track 1), corresponding to the molecular weight of the protein, determined previously to be 39 kDa (11). The truncated *Vh*ChiP channel migrated faster in the gel (Fig. 1*A*, track 2), consistent with its predicted molecular weight of 36.4 kDa.

X-ray diffraction data of ligand-free truncated *Vh*ChiP were indexed, integrated, and scaled at a resolution of 2.40 Å in monoclinic space group *C*121, with a single trimer per asymmetric unit. The structure of the ligand-free truncated *Vh*ChiP was determined by molecular replacement (MR), using the refolded *Vh*ChiP (PDB ID: 5MDO) as a structural template. The final model of the ligand-free truncated *Vh*ChiP was refined to *R*_work_ and *R*_free_ values of 18.0% and 22.6%, respectively. X-ray diffraction data from truncated *Vh*ChiP cocrystallized with chitohexaose were obtained to a resolution of 2.75 Å. The data were processed in the triclinic P1 space group with two trimers per asymmetric unit. The structure of the ligand-bound truncated *Vh*ChiP was solved by MR, using the ligand-free truncated *Vh*ChiP structure as the template. The structure of chitohexaose was retrieved from the structure of *Vh*ChiP in complex with chitohexaose (PDB ID: 5MDR) and placed in the electron density map. The refined model for the chitohexaose-truncated *Vh*ChiP complex had refinement statistics *R*_work_ and *R*_free_ of 18.1% and 22.3%, respectively. The crystal structures of the ligand-free truncated *Vh*ChiP and the chitohexaose-bound truncated *Vh*ChiP were deposited under the PDB IDs of 7EQM and 7EQR, respectively. The details of crystallographic statistics of both structures are presented in Table 1. Inspection of the structures revealed that both truncated structures existed as intact trimers (Fig. 1*B*), indicating that the *N*-plug truncation did not affect the assembly of the protein. The trimeric subunits were held together by loops L2 (blue), while the longest loop L3 (green) protruded into the channel lumen, and loop L8 (red) covered the entrance of the protein pore on the extracellular side. The ligand-free truncated channel was open (Fig. 1*C*, top view and side view), as in the structure of the *in vitro*–refolded *Vh*ChiP (PDB ID: 5MDO, ref. (20)). We observed electron density for the sugar ligand in all six protein chains (two trimeric molecules). However, the complete electron density map of six GlcNAc rings of chitohexaose was observed only in chains B and F, while the electron densities of the sugar molecules in chains A, C, D, and E were partial, missing mainly the areas assigned to GlcNAc-1 (nonreducing end) and GlcNAc-6 (reducing end) of the sugar chain. For the structures of chains B and F, (Fig. 1*D*, upper panel), the sugar chain was fully extended inside the pore, in a similar manner to chitohexaose found in the native structure. The six GlcNAc rings of the sugar chain extended throughout the affinity sites 1-6 from the periplasmic side toward the extracellular side (Fig. 1*C*, lower panel).

**Table 1:** Data collection and refinement statistics of the truncated *Vh*ChiP channels in the absence and presence of chitohexaose

PDB ID	Apo truncated *V*hChiP 7EQM	Truncated *Vh*ChiP+chitohexaose
		7EQR
Wavelength	0.97	0.97
Resolution range	19.91–2.50 (2.59–2.50)	19.82–2.75 (2.85–2.75)
Space group	C 1 2 1	P 1
Unit cell		
a, b, c (Å)	105.2, 151.4, 95.8	58.0, 131.2, 136.6
α, β, γ (°)	90, 111.1, 90	65.8, 87.8, 86.9
No. of asymmetric unit	1 trimer	2 trimers
Total reflections	200,430	500,859
Unique reflections^a^	43,100 (3248)	85,921 (6047)
Completeness (%)	89.2 (67.7)	90.1 (63.9)
Mean I/sigma(I)	12.2 (2.9)	9.9 (1.8)
Multiplicity	3.6 (3.1)	5.1 (3.4)
Wilson B-factor	31.8	42.5
R_meas_	0.119 (0.397)	0.188 (0.730)
R_pim_	0.061 (0.213)	0.081 (0.345)
CC_1/2_	(0.864)	(0.776)
Reflections used in refinement	43,094 (3248)	85,896 (6047)
Reflections used for R_free_	2137 (166)	4784 (294)
R_work_	0.180 (0.197)	0.181 (0.211)
R_free_	0.226 (0.292)	0.223 (0.275)
Number of nonhydrogen atoms	8149	16,450
Macromolecules	7731	15,462
Ligands	63	718
Solvent	355	304
Protein residues	993	1986
RMS (bonds) (Å)	0.007	0.009
RMS (angles) (˚)	0.87	1.03
Ramachandran plots		
Favored (%)	97.16	95.95
Allowed (%)	2.63	3.95
Outliers (%)	0.2	0.1
Rotamer outliers (%)	0.13	5.82
Clash score	3.56	12.36
Average B-factor (Å^2^)	33.0	43.2
Macromolecules	32.9	42.6
Ligands	38.5	57.6
Solvent	34.2	41.0

Values in parentheses are for the outer resolution shell.

### The structures of closed and open sugar-bound states of *Vh*ChiP differ only in the position of the side chain of Tyr349

**Figure 2:**
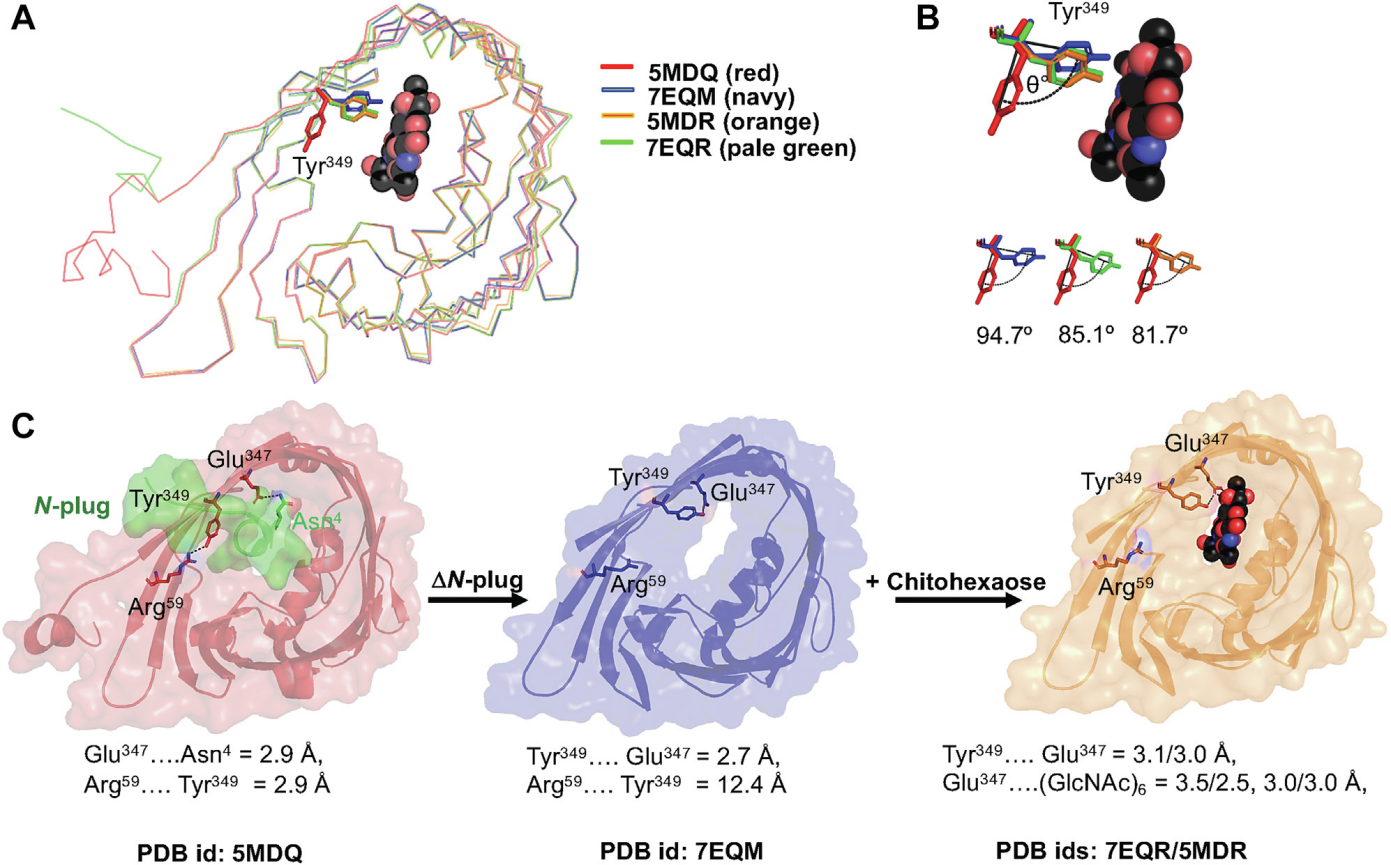
**Structural comparison of truncated *Vh*ChiP with the native *Vh*ChiP structures.***A*, superimposition of C_α_ backbone of *Vh*ChiPs: 7EQM (ligand-free truncated, open channel) and 7QER (sugar-bound truncated) from this study, and 5MDQ (ligand-free WT, plugged channel) and 5MDR (sugar-bound WT) from the previous study, (20). *Green* represents the N-plug of 5MDQ. Chitohexaose is shown as *sphere. B*, the deviation of angle in Tyr^349^ in the open channel or in the sugar-bound channels when compared with the plugged channel (5MDR). *C*, distance and interactions of the side chain of Tyr^349^ in each *Vh*ChiP variant with its neighboring side chains. Hydrogen bonds are typically ≤ 3.5 Å.

Superimposition of the *C*_α_ backbone of ligand-free truncated *Vh*ChiP (PDB ID: 7EQM) and chitohexaose-bound truncated *Vh*ChiP (PDB ID: 7EQR) with the unplugged refolded *Vh*ChiP (PDB ID: 5MDO) and sugar-bound *Vh*ChiP (PDB ID: 5MDR) yielded RMSD values for 340 *C*_α_ atoms of 0.22 and 0.28 Å (Fig. 2*A*), respectively, suggesting considerable similarity in their rigid backbones. Nevertheless, a significant movement of the side chain of Tyr^349^ on the internal surface of the β-barrels was apparent between the closed and open channels (Fig. 2*A*). In the plugged WT channel (PDB ID: 5MDQ), the side chain of Tyr^349^ was adjacent to the channel wall (red stick), while this residue pointed toward the middle of the pore in the open truncated channel (PDB ID: 7EQM, navy stick) and in both WT and truncated channels occupied by chitohexaose (PDB IDs: 5MDR, orange stick and 7EQR, pale green stick) (Fig. 2*A*). The angular shift from the Tyr^349^ side chain position in 5MDQ was 94.7° (7EQM), 85.1° (5MDR), and 81.7° (7EQR), respectively (Fig. 2*B*). The Tyr^349^ side chain in the plugged structure (Fig. 2*C*) was located close to the channel wall and formed a hydrogen bond with the side chain of the nearby (2.9 Å) residue Arg^59^ on strand B2. In contrast, the side chain of Tyr^349^ in the open and sugar-bound structures (Fig. 2*C*, 7EQM, 7EQR, and 5MDR) rotated away from Arg^59^ in the opposite direction and formed an H-bond with the side chain of Glu^347^ instead (approx. 2.7 Å).

### *N*-terminal truncation caused the substantial loss of H-bonds around the central affinity sites

**Figure 3:**
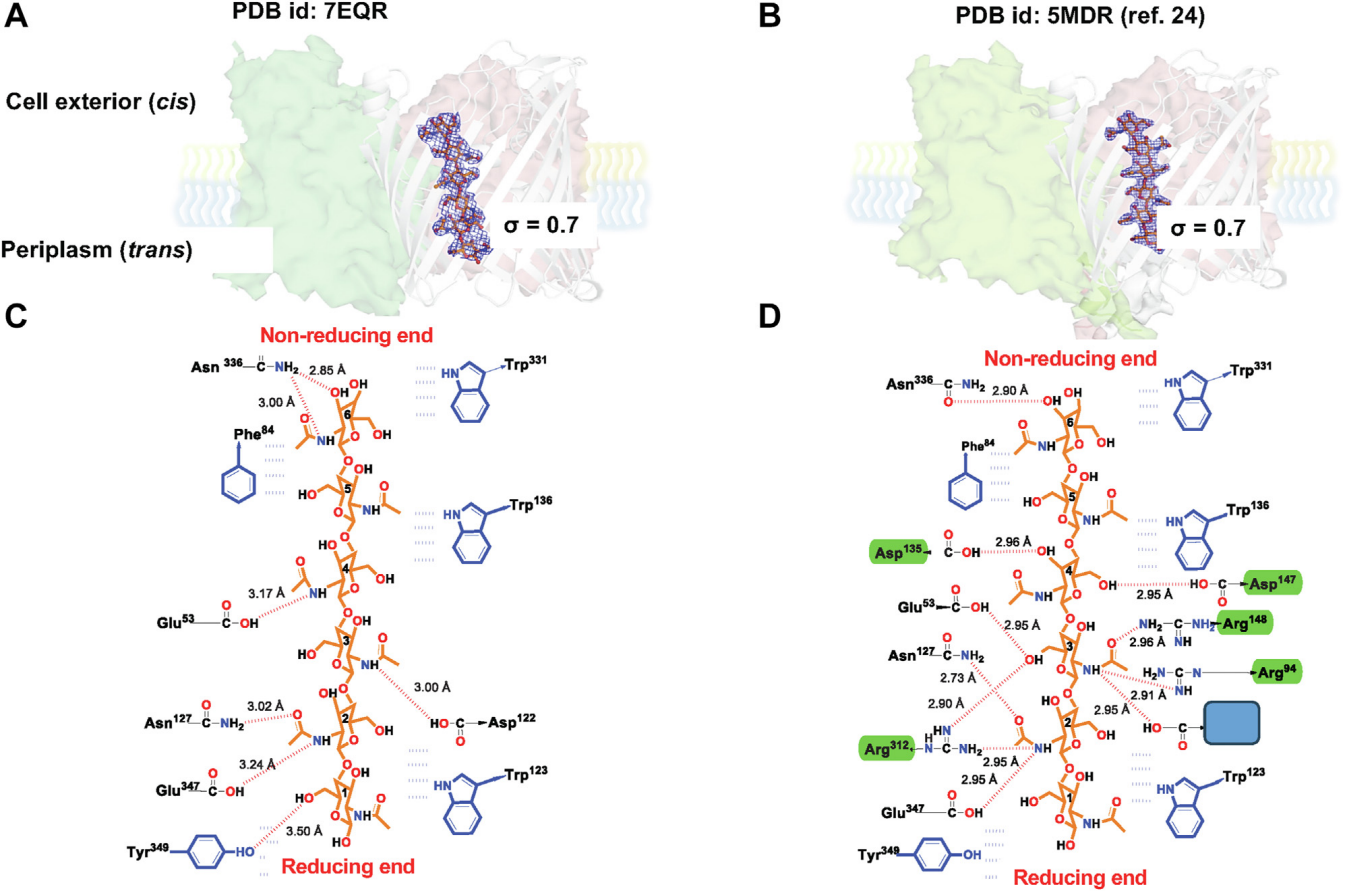
**LIGPLOT analysis of *Vh*ChiP binding to chitohexaose.** The affinity sites are designated with numbers 1 to 6. *A* and *B* show the electron density map of chitohexaose in one of the monomers of the truncated channel (PDB ID: 7EQR) and WT channel (PDB ID: 5MDR; ref. (20)), respectively, with the OMIT maps of chitohexaose contoured at sigma = 0.7. *C* and *D* show the detailed interactions of chitohexaose with the pore-lining residues. Sugar backbones are shown as 2D with atoms colored *orange* for C, *red* for O, *black* for H, and *blue* for N. Hydrogen bonds (*green*) between the residues and the GlcNAc rings at the affinity sites 2 to 4 are missing in the truncated channel. *Red broken lines* represent hydrogen bonds. *Blue residues* form hydrophobic interactions. Residues that are involved in an extra H-bond in (*D*) relative to (*C*) are labeled with a *green background*.

The crystal structure of the truncated *Vh*ChiP in complex with chitohexaose (PDB ID: 7EQR) contained two trimers per asymmetric unit, and two chains had sufficient electron density to fit chitohexaose inside the channel. The electron density of four GlcNAc rings was strong at the affinity sites 2 to 5 but weak at affinity sites 1 and 6 (Fig. 3*A*). As a result of weak interactions, the quality of the electron density of chitohexaose in the truncated channel was poorer than that of chitohexaose in the WT channel (PDB ID: 5MDR) (Fig. 3*B*). We observed that each of the trimeric β-barrels contained one molecule of chitohexaose inside the channel lumen (Fig. 3*C*) in which the reducing end sugar (GlcNAc-1) made π–π interactions with the plane of the aromatic side chains of Trp^123^ and Tyr^349^. One weak hydrogen bond was observed between the side chain of Tyr^349^ and the *N*-acetamido group of GlcNAc-1 (3.5 Å).

At affinity site 2, two weak hydrogen bonds were observed, one between the carboxylate side chains of Asp^127^ and the *O*-acetamido group of GlcNAc-2 (3.02 Å) and another between the side chain of Glu^347^ and the *N*-acetamido group of GlcNAc-2 (3.24 Å). The plane of GlcNAc-2 also stacked against the plane of Trp^123^. For affinity sites 3 and 4, only one hydrogen bond was seen at each site, in which the carboxyl side chain of Asp^122^ bonded with the *N*-acetamido group of GlcNAc-3 (3.00 Å) and of Glu^53^ with the *N*-acetamido group of GlcNAc-4 (3.17 Å). GlcNAc-4 also made hydrophobic interactions with Trp^136^, while GlcNAc-5 made hydrophobic interactions with the side chains of Phe^84^ and Trp^136^ and GlcNAc-6 with Phe^84^ and Trp^331^, respectively. GlcNAc-6 also formed two hydrogen bonds with the side chain of Asn^336^. The crystal structure of the WT *Vh*ChiP showed clear electron density for chitohexaose with high-resolution diffraction data (1.90 Å), as well as high-binding affinity (20). Although the hydrophobic interactions at affinity site 1 (GlcNAc-1/Trp^123^/Tyr^349^) and affinity site 5 (GlcNAc-5/Phe^84^/Trp^136^) and site 6 (GlcNAc-6/Phe^84^/Trp^331^) were similar, more hydrogen bonds were observed in the WT channel (Fig. 3*D*) than in the truncated channel (Fig. 3*C*). Five hydrogen bonds were missing in the truncated structure (Fig. 3*D*, residues highlighted in green), including the *N*-acetamido group of GlcNAc-2/Arg^312^ (2.95 Å) and GlcNAc-3/Arg^94^ (2.91 Å), the *O*-acetamido groups of GlcNAc-3/Arg^148^ (2.96 Å) and Glu^53^/*O*-acetamido of GlcNAc-3 (2.95 Å), the *O*-acetamido of GlcNAc-3 Arg^312^/O- (2.90 Å) and the *O*-acetamido of GlcNAc-4/Asp^135^ (2.96 Å)/Asp^145^ (2.95 Å). A summary of interactions at each affinity site of both channels with chitohexaose is given in Table S1.

### Single-channel electrophysiology reveals the *N*-terminal truncation caused the substantial loss in the binding affinity

Single-channel analysis using the black lipid membrane (BLM) reconstitution technique was employed to examine the effects of the Δ1-9 truncation on pore conductance of *Vh*ChiP (Fig. S1). The WT *Vh*ChiP could insert into artificial lipid membranes and remained fully open with occasional gating during a 2-min recording time under the given conditions (1 M KCl in 20 mM Hepes, pH 7.4) (Fig. S1*A*). Histogram analysis of a total of 57 stepwise multiple insertions suggested an average single-channel conductance of 2.05 ± 0.2 nS (Fig. S1*B*), a value comparable to that of the OM-expressed *Vh*ChiP (1.8 ± 0.3 nS) reported previously (11). The truncated channel inserted relatively quickly into the membrane and remained more stably open, with no gating (Fig. S1*C*). The average channel conductance of truncated *Vh*ChiP was 2.0 ± 0.4 nS, estimated from histogram analysis of 60 insertions (Fig. S1*D*), similar to that of WT *Vh*ChiP.

**Figure 4:**
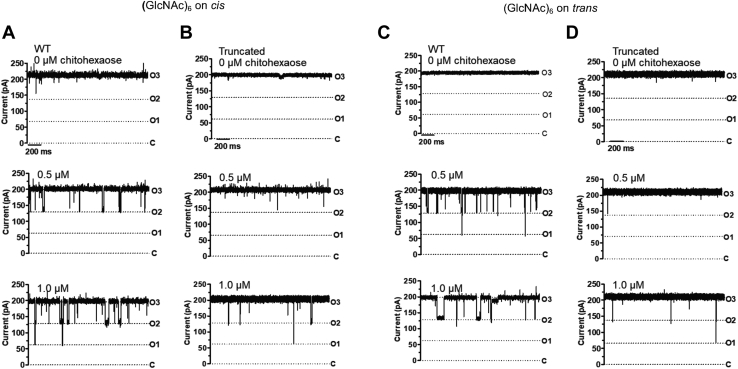
**Sugar-channel interaction studies by black lipid membrane (BLM) technique.** The BLM experiments were carried out by adding chitohexaose on the *cis* side to (*A*) WT *Vh*ChiP, (*B*) truncated *Vh*ChiPs or *trans* side of (*C*) WT *Vh*ChiP and (*D*) truncated *Vh*ChiP reconstituted in lipid membranes. Both sides of the chamber were bathed with 1 M KCl in 20 mM Hepes, pH 7.4. The purified *Vh*ChiP (20 μg mL^−1^, 1 μl) was always added on the *cis* side. Both channels were exposed to different concentrations of chitohexaose (0–1 μM, but only traces at 0, 0.5, and 1.0 μM are shown). States of the channel in lipid membrane: O3, trimeric opening; O2, monomeric closure; O1, dimeric closure; and C: trimeric closure. Current traces were recorded continuously at 25 ± 1 °C at two potentials, ±100 mV (here only +100 mV is shown) for 2 min.

The effect of the *N*-plug truncation on the binding affinity was investigated by single-channel analysis. Figure 4, *A* and *B* show ion traces from WT and truncated *Vh*ChiP, respectively, observed with an applied potential of +100 mV in 1 M KCl electrolyte and with different concentrations of chitohexaose (Fig. 4) added to the *cis* side of the chamber. It was clear that the sugar interacted strongly with the WT channel, causing temporary occlusion of ion flow in a concentration-dependent manner. In Figure 4*A*, we observed transient falls in the ionic current, mainly from the fully open (state O3) to one monomeric closure (state O2) as the sugar concentration was increased from 0 to 0.5 μM, and only occasionally did the ionic current fall further, with dimeric closure (state O1) when the concentration of chitohexaose was increased to 1.0 μM. The average residence time (τ_c_) for the sugar molecule inside the channel was 3.7 ± 0.3 ms, and the estimated on-rate (*k*_on_) is 56 ± 10 × 10^6^ M^-1^s^-1^. In contrast, rare and fast-blocking events were observed for the truncated channel at the same concentration range of the sugar and falls in current were only less frequently observed. The average residence time estimated for the sugar inside the truncated channel is about 2.4 ms and the on-rate is 12 × 10^6^ M^−1^s^−1^. The binding constant (*K*) estimated for the WT is 250,000 ± 50,000 M^−1^, which is 8.3-fold greater than that for the truncated channel (30,000 ± 9000 M^−1^) (Table 2). Similar results were observed with sugar addition on *trans* side (Fig. 4, *C* for WT and *D* for truncated), the kinetic values being slightly different.

**Table 2:** Kinetic parameters of the truncated *Vh*ChiP titrated with the synthesized *N*-plug peptide

*Vh*ChiP	Sugar	*N*-plug	*k*_on_ × 10^6^ (M^−1^s^−1^)	τ_c_ (ms)	*k*_off_ × 10^3^ (s^−1^)	*K* (M^−1^)
WT	*cis*	-	68 ± 1.4	3.7 ± 0.4	0.27 ± 0.4	250,000 ± 50,000
	*trans*	-	50 ± 1.2	4.7 ± 0.5	0.21 ± 0.5	240,000 ± 70,000
Truncated	*cis*	-	5.6 ± 0.3	2.0 ± 0.3	0.50 ± 0.1	11,000 ± 3000
	*trans*	-	6.8 ± 0.5	3.3 ± 0.5	0.30 ± 0.1	23,000 ± 5000
Truncated	*cis*	*trans*	56 ± 0.1	4.5 ± 1.0	0.22 ± 0.01	255,000 ± 10,000
	*cis*	*cis*	-	-	-	n.d.

The equilibrium binding constant (*K*, M^−1^) is estimated from Equation 1, which is derived from the relative reduction of the average single channel conductance when the channel was titrated with different concentrations of chitohexaose. The on-rate (*k*_on_, M^−1^ s^−1^) is given by *k*_on_ = *K* · *k*_off_ and the off-rate (*k*_off,_ s^−1^) for the single trimeric molecule of *Vh*ChiP channel and its mutant by titration with chitohexaose from the relationship *k*_off_ = 1/*τ*_c_.

n.d., nondetectable blocking events.

**Figure 5:**
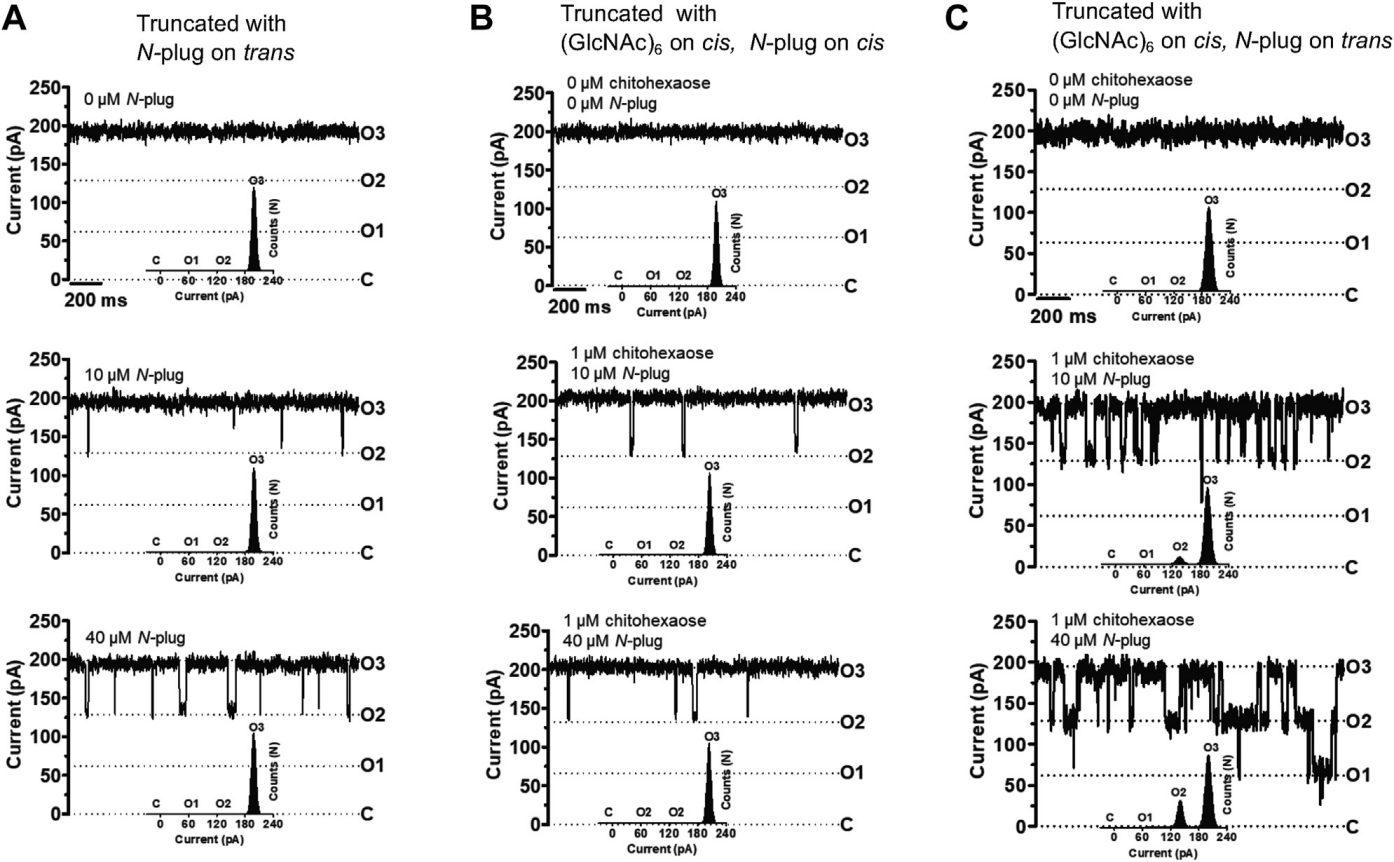
**The effects of *N*-plug peptide binding on sugar–channel interactions.** Single-channel experiments were carried out with the truncated channel as follows; (*A*) no sugar addition, varied concentrations of the *N*-plug peptide added on the *trans* side, (*B*) fixed concentration of chitohexaose (1 μM) on the *cis* side, varied concentrations of the *N*-plug peptide added on the *cis* side (10 and 40 μM); (*C*) fixed concentration of chitohexaose (1 μM) on the *cis* side, varied concentrations of the *N*-plug peptide added to the *trans* side. Current traces were recorded continuously at 25 ± 1 °C at ±100 mV (here only +100 mV are shown) for 2 min for each dataset.

The *N*-plug peptide corresponding to the first nine residues (DGANSDAAK) inside the protein pore was synthesized and added on either *cis* or *trans* side of the sample cell partitioned by the lipid bilayer, and its effects on the binding kinetics of sugar–channel interactions were investigated. Figure 5 shows the control traces without sugar addition on either side but with *N*-plug peptide added on the *trans* side at various concentrations. Under the applied potential of +100 mV in 1 M KCl, 20 mM Hepes, pH 7.4, rare and short-lived blocking events were observed when the truncated channel was exposed to the *N*-plug peptide at 10 and 40 μM (Fig. 5*A*). Similar results were obtained when the *N*-plug peptide was added on the *cis* side without sugar (data not shown) and with both chitohexaose (1 μM) and the *N*-plug peptide (10 and 40 M) on *cis* side (Fig. 5*B*). The results were markedly different with the addition of chitohexaose on the *cis* side and the *N*-plug peptide on the *trans* side. Strong blocking events at a monomeric level from state O3 (fully open) to O2 (one monomer closure) were observed with 10 μM *N*-plug peptide added. When the concentration of the peptide was increased to 40 μM, blocking events in the monomeric closure (state O2) were much more frequent, along with sporadic blocking in the dimeric closure (state O1) (Fig. 5*C*). With the addition of 40 μM *N*-plug peptide, overlapping blocking events of states O2 and O1 were observed, allowing more than one sugar molecule to remain, at the same time, inside the protein channel (Fig. 5*C*, bottom trace).

Table 2 shows the kinetic values for chitohexaose interactions with *Vh*ChiP variants that were obtained from noise analysis of single-channel measurements. The equilibrium binding constant (*K*, M^−1^) estimated for chitohexaose additions on the *cis* and *trans* side of lipid bilayer reconstituted with the WT channel were similar (250,000 and 240,000 M^−1^, respectively). For the truncated channel, the *K* values for chitohexaose additions on the *cis* and *trans* sides were 11,000 M^−1^ and 23,000 M^−1^, respectively. When chitohexaose was added on the *cis* and *N*-plug on the *trans* side, the *K* value returned to 255,000 M^−1^, the value for chitohexaose–WT channel interactions. In contrast, the *K* value for the addition of both sugar and the *N*-plug peptide on the *cis* side was incalculable, as there were insufficient blocking events.

### The decrease in binding affinity of the truncated channel was confirmed by ITC

**Figure 6:**
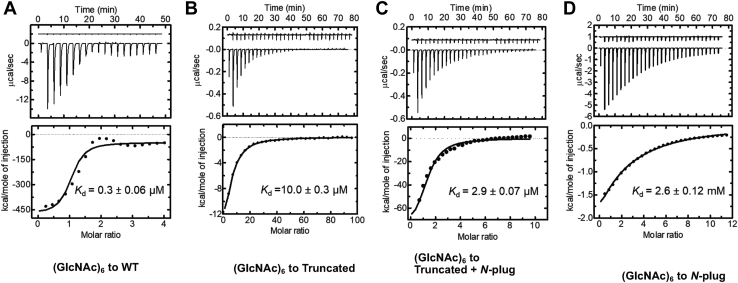
**Binding study by isothermal titration microcalorimetry.** Titration of chitohexaose into (*A*) WT *Vh*ChiP, (*B*) truncated *Vh*ChiP, (*C*) truncated *Vh*ChiP mixed with the *N*-plug peptide, and (*D*) the synthesized *N*-plug peptide. *Top panels* are thermograms and *bottom panels* are the corresponding heat integrations with theoretical fits using a nonlinear one-site binding function, available in MicroCal PEAQ-ITC analysis software. ITC experiments were performed at 25 °C. The data were corrected for heats of dilution obtained by titration of chitohexaose into buffer (control data offset by +10 μcal.sec^−1^, *top traces*). Data presented are mean ± SD from the same set of experiments, which were carried out in triplicate. ITC, isothermal titration microcalorimetry.

The effects of *N*-plug truncation on the binding affinity of *Vh*ChiP were investigated using ITC. The ITC measurements yielded negative thermograms, indicating that all binding events occurred exothermically. Figure 6, top panels show ITC thermograms, and the bottom panels show the corresponding heat integration plotted against the molar ratio, with curve fitting using a one-site binding function. Chitohexaose titration into WT *Vh*ChiP (Fig. 6*A*) yielded an equilibrium dissociation constant (*K*_d_) of 0.3 ± 0.1 μM and a stoichiometry of 3.2 ± 0.1. Titration of chitohexaose into the truncated channel yielded a *K*_d_ of 10 ± 0.3 μM and a stoichiometry of 2.8 ± 0.1 (Fig. 6*B*), while titration of chitohexaose with the truncated *Vh*ChiP mixed with the *N*-plug peptide (Fig. 6*C*) yielded a *K*_d_ of 2.9 ± 0.07 μM and a stoichiometry of 2.3 ± 0.2. Chitohexaose was shown to interact with the *N*-plug peptide with a *K*_d_ of 2.6 ± 0.12 mM and a stoichiometry of 1.0 ± 0.02. A summary of the ITC experiments and data appears in Table 3.

**Table 3:** Binding parameters for sugar–*Vh*ChiP interactions by isothermal titration calorimetry

	Chitohexaose titration				
	WT	Truncated *Vh*ChiP	Truncated *Vh*ChiP + *N*-plug peptide	*N*-plug peptide	D1A mutant
Substrate (mM)	0.6	5.0	1.0	30	0.8
Protein (μM)	20	20	20	-	20
*N*-plug (μM)	-	-	5	500	-
*n* (site)	3.2 ± 0.1	2.8 ± 0.1	2.3 ± 0.2	1.0 ± 0.02	3.0 ± 0.1
*K*_d_ (μM)	0.3 ± 0.06	10.0 ± 0.3	2.9 ± 0.07	2600 ± 120	1.2 ± 0.2

### SMD simulations confirmed that the sugar molecule induced the N-plug ejection

**Figure 7:**
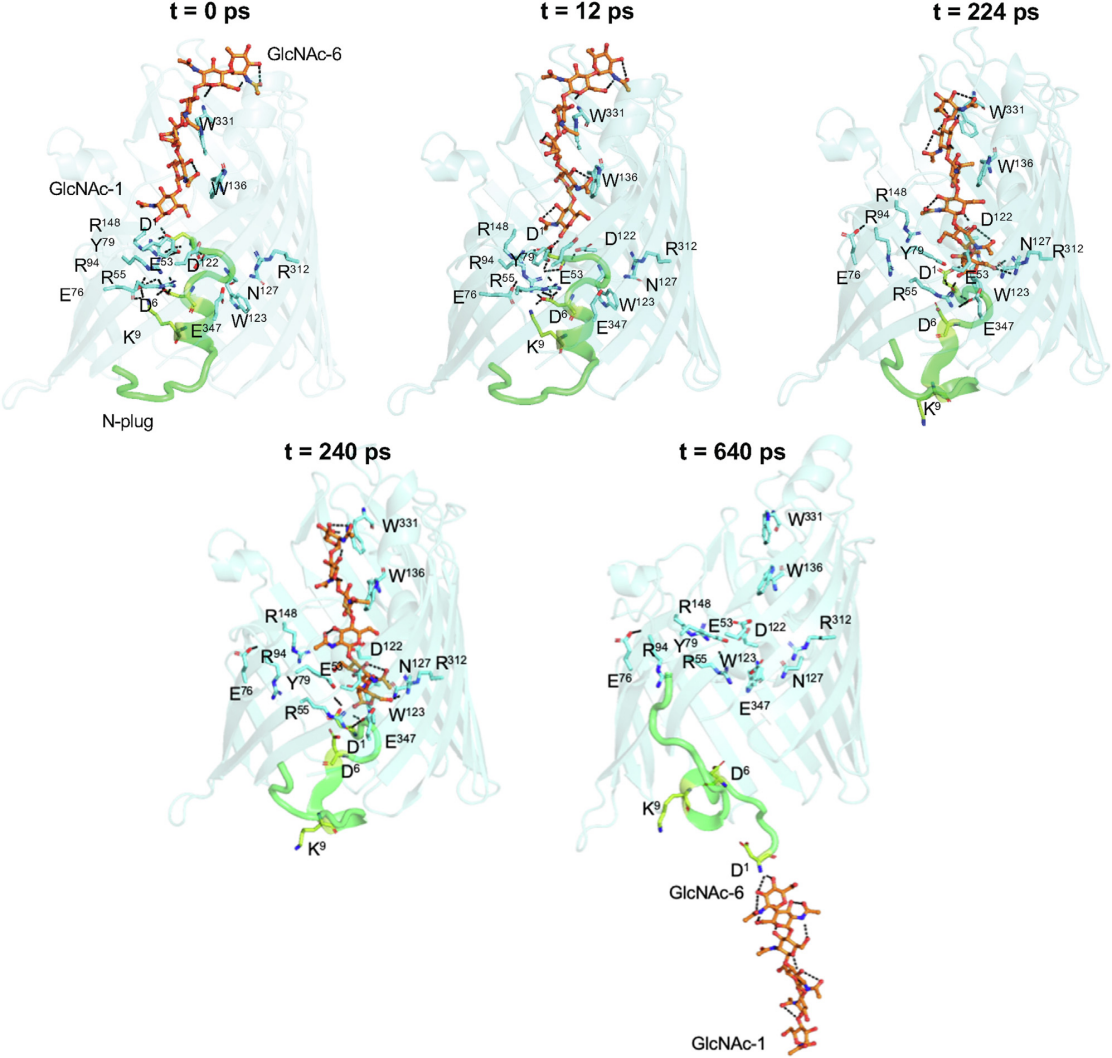
**Main snapshots of sugar movement into the plugged pore obtained from SMD simulation from 0 to 700 ps.** At t = 0, the sugar chain is stretched on top of the *Vh*ChiP pore. At t = 12 ps, the reducing end of GlcNAc-1 has formed a strong H-bond with Asp^1^. At t = 224 ps, sugar is displaced and Asp^6^ detached from the channel wall. At t = 240 ps, Lys^9^ is detached from Glu^76^. Trp 123(part of L3) and Trp^331^ (part of L8) are displayed to indicate insignificant change inside chain movement of the pore-lining residues during sugar insertion. At t = 640 ps, the *N* terminus has been ejected and the sugar released. SMD, steered molecular dynamics.

To address the molecular mechanism of translocation, SMD simulations were used to observe sugar movement through the pore. The plugged form of the OM-expressed *Vh*ChiP (PDB ID: 5MDQ) was chosen as the starting point for molecular docking as it contained the *N*-plug inside the pore. Our previous applied force molecular dynamics (MD) simulations suggested that the *N*-plug was stable inside the pore in the absence of sugar (20), and a similar result was observed with our initial SMD simulation performed with the plugged channel without sugar. The snapshots of the first frame and the last frame were superimposed with RMSD of 1.3 with no ejection of the *N*-plug, suggesting that the *N*-plug was stable over the period of the simulation (Fig. S2*A*). To observe sugar movement inside the channel, SMD simulation was carried out with the plugged pore, the sugar substrate (chitohexaose) being placed on the exterior of the protein pore and the three residues Ala^143^-Gln^146^, which are part of loop L3, fluctuated slightly, presumably through the motion of the *N*-plug (20). However, our SMD simulations did not reveal much movement of L3. We further inspected the crystal structures of the plugged channel without (5MDQ) and with sugar (5MDR) (Fig. S2*B*), which showed no side chain movement upon sugar insertion, and the distance from the most extended side chain of Tyr147 (13.1 Å) was too far for the *N*-plug to bond with these residues (Fig. 3*C*). The main observation was that the reducing end of the sugar chain inserted into the protein pore overlapped with the tail of the *N*-plug. Superimposition of the sugar molecule in the structure of 5MDR and 7EQR (Fig. S3*A*) showed the sugar chain interacting with polar residues that mostly bind the *N*-plug (Fig. S3*B*). The interactions of the *N*-plug with these residues were disrupted during sugar protrusion. Figure 7 shows five snapshots, demonstrating considerable motion of the sugar molecule and the *N*-plug inside the protein pore. Overall, SMD simulation suggested a displacement mechanism in which subsequent interactions of the reducing sugars with the tail of the *N*-plug assist sugar translocation (20).

At t = 0, the sugar chain occupied the extracellular side of the protein pore, with the reducing end sugar ring (GlcNAc-1) located 4.5 Å away from the *N*-terminal residue (Asp^1^) of the *N*-plug. However, (GlcNAc)_6_ did not interact with any of the residues lining the pore. Asp^1^ was inserted deepest inside the bottom half of the protein pore, stabilized by H-bonds with the cluster of adjacent pore-lining residues: Glu^53^, Tyr^79^, Tyr^118^, Asp^122^, Asn^127^, Arg^148^, Arg^312^, and Glu^347^. Additionally, Asp^6^ and Lys^9^ in the *N*-plug formed other clusters of H-bonds, with Arg^55^-Asp^6^-Lys^9^-Arg^94^ and Asp^6^-Lys^9^-Glu^76^, respectively.

At t = 12 ps, the chitohexaose chain moved down the pore toward the constriction zone, where the reducing end of GlcNAc-1 formed a strong H-bond with Asp^1^ of the *N*-plug, while Asp^1^ remained bonded to Glu^53^ and Tyr^79^.

At t = 224 ps, the sugar chain moved further down the channel lumen, creating a constraint to the *N*-plug and subsequently caused Asp^6^ to detach from Arg^55^ and Arg^94^.

At t = 240 ps, the sugar completely displaced the *N*-plug, leaving it ready to be ejected from the protein pore, while GlcNAc-1 remained attached to Asp^1^. At this time point, Lys^9^ also detached from the channel wall.

At t = 640 ps, the *N*-plug was completely ejected, while interactions of the reducing end sugar rings (GlcNAc-1 and GlcNAc-2) with Asp^1^ (side chain), Gly^2^ (main chain), and Asp^6^ (side chain) of the *N*-plug through a H-bond network may provide additional force for the sugar molecule to be translocated. For more details, the MD simulation movie is attached in the Supplementary (MV1).

### Site-directed mutation of Asp^1^ revealed that the sugar interacted with the tail of the *N*-plug through *H*-bonding

**Figure 8:**
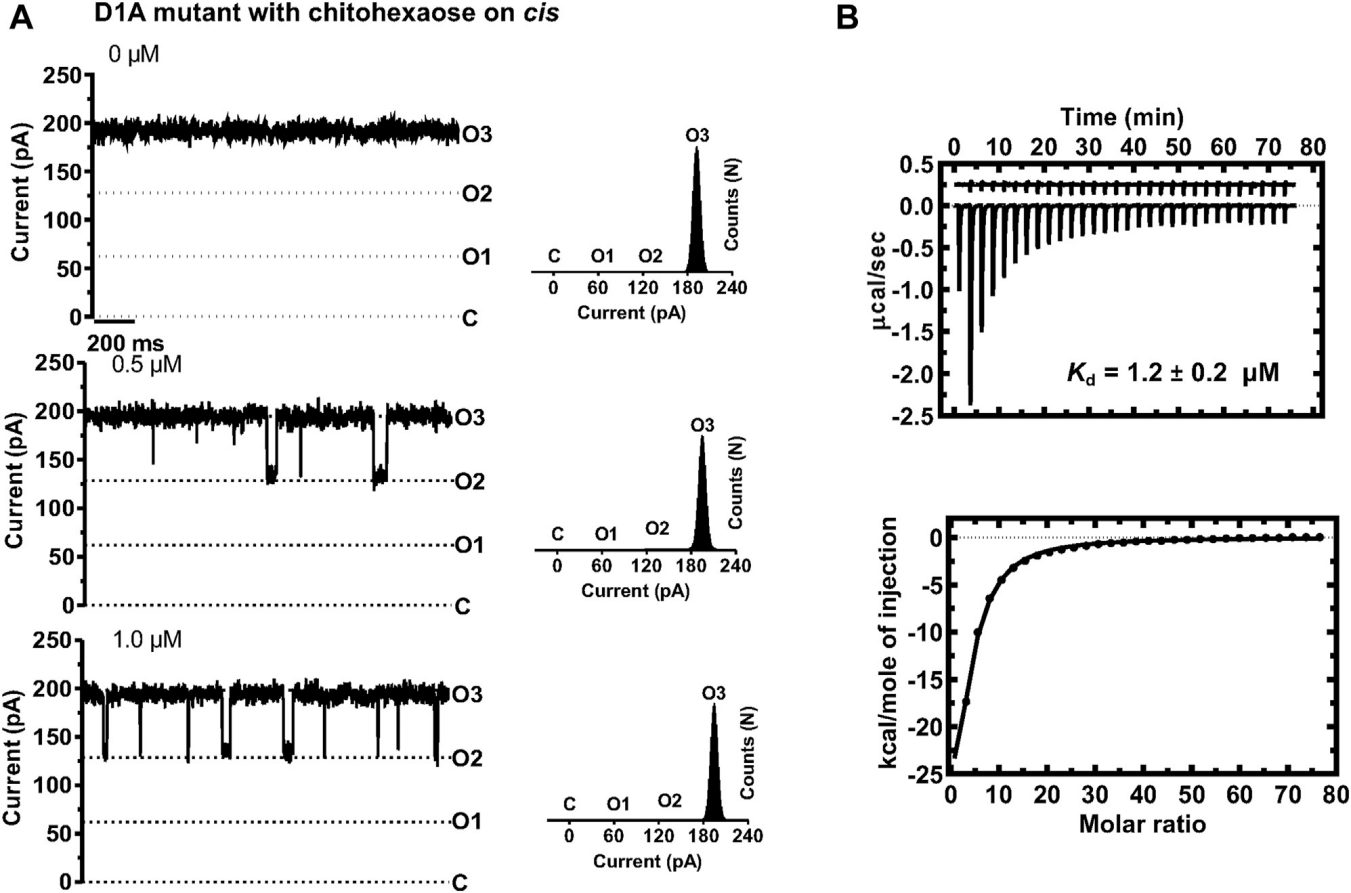
**Effects of the Asp**^**1**^**mutation on the binding affinity of *Vh*ChiP for chitohexaose.** Asp^1^ was mutated to Ala, generating the D1A mutant. *A*, reconstitution of the D1A mutant into *black* lipid membrane and occlusion of ion flow upon the addition of chitohexaose at different concentrations to the *cis* side of the lipid bilayer. Single-channel measurement was carried out as described for the WT and the truncated *Vh*ChiPs. Each dataset was acquired at an applied potential of +100 mV in 1 M KCl, 20 mM Hepes, pH 7.4. Data were acquired over 2 min. *Right-hand panels* show the corresponding histogram analysis. *B*, binding studies by ITC are as follows: a thermogram of chitohexaose titration into the D1A channel is shown in the *upper panel*, while heat integration with nonlinear curve fitting is shown in the *lower panel*. The state of the channel in lipid membrane under applied voltages is shown as O3 for fully open, O2 for one monomeric closure, O1 for dimeric closure, and C for fully closed. ITC, isothermal titration microcalorimetry.

To investigate the important role of the *N*-plug in sugar translocation, we further mutated Asp^1^ to Ala and conducted electrophysiological and ITC studies of the interaction of the D1A mutant with chitohexaose. Figure 8*A* shows that all trimers of the D1A mutant were stably open in artificial lipid membranes at the applied potential of ±100 mV. The average conductance of the fully open channel was 200 ± 0.1 nS, which is slightly larger than that of the unmutated channel (Fig. 4*A*). On titration with chitohexaose on the *cis* side at concentrations of 0 to 1.0 μM, less frequent monomeric blockages of the ionic current were observed, and the binding curve yielded a *K* value of 100,000 ± 20,000 M^−1^, which was 2.5-fold less than that for the WT channel. Figure 8*B* (upper panel) shows an exothermic thermogram from ITC experiments, obtained by adding chitohexaose to the D1A mutant. Analysis of the fitted theoretical curve of heat integration (lower panel) yielded a *K*_d_ value of 1.2 ± 0.2 μM (Table 3).

## Discussion

Porins are protein channels found on the OM of Gram-negative bacteria and have a major role in controlling membrane permeability, preventing the entry of noxious compounds, and allowing the entry of nutrient molecules that are required for cell growth and function (21). *Vh*ChiP is a chitooligosaccharide-specific channel found in the OM of *V. campbellii* (formerly *V. harveyi*) strain American Type Culture Collection BAA 1116. The channel can interact with chitohexaose with high affinity, the binding constant (*K*) is 250,000 to 500,000 M^−1^ ((17, 22), and this study). This value is at least one or two orders of magnitude greater than that reported for other sugar-specific channels, for instance maltoporin (LamB) for maltohexaose (13,600 M^−1^) (23), CymA for cyclodextrin (31,300 M^−1^) (24), and ScrY for sucrose (80 M^−1^) (25). The strong binding reflects the remarkable efficiency of the *Vh*ChiP channel in recognizing its substrate and coordinating chitooligosaccharide transport. In comparison, we reported the functional characterization of a ChiP homolog (*Ec*ChiP) from *E. coli* (26, 27) and found that *Ec*ChiP contained only one subunit, with a single channel conductance (0.5 ± 0.05 nS) only one-third of that in the trimeric *Vh*ChiP (2.0 ± 0.1 nS). Kinetic evaluation from single-channel analysis gave a *K* value for chitohexaose of 50,000 M^−1^ for *Ec*ChiP, which is about 10-fold less than that of *Vh*ChiP for the same sugar. The data indicated the role of *Vh*ChiP in meeting the physiological requirement of *V. campbellii* in using chitin as a primary carbon source and a lesser demand by *E. coli*, which uses chitin only as an alternative energy source.

The structural details suggested that the OM-expressed *Vh*ChiP trimers (PDB ID: 5MDQ) were occluded by the *N*-plug located on the periplasmic side, while these plugs were ejected in the open channel (PDB ID: 5MDO) and the sugar-bound channel (PDB ID: 5MDR), suggesting that the *N*-plugs controlled the closed/open dynamics of the protein channel (20). The existence of *N*-plugs was a unique characteristic of the previously described TonB-dependent transporters (28) and oligosaccharide-specific channels (CymA) from *Klebsiella oxytoca* (29) but was not observed in nonspecific porins or other sugar-specific channels. The existence of the *N*-plug that acts as a ligand-expelled gate to control channel opening/closing was previously reported for TonB-dependent transporters (28) and cyclodextrin-specific transporter (CymA) (29). For CymA, the crystal structure in complex with cyclodextrin and SMD simulations suggested that bulky cyclodextrin (α-CD) changes its orientation inside the pore from a flat to a linear shape as the molecule travels through a narrow pore and that this disrupts charge–charge interactions between the *N*-plug and the channel wall, causing the release of the *N*-terminal plug from the pore.

**Figure 9:**
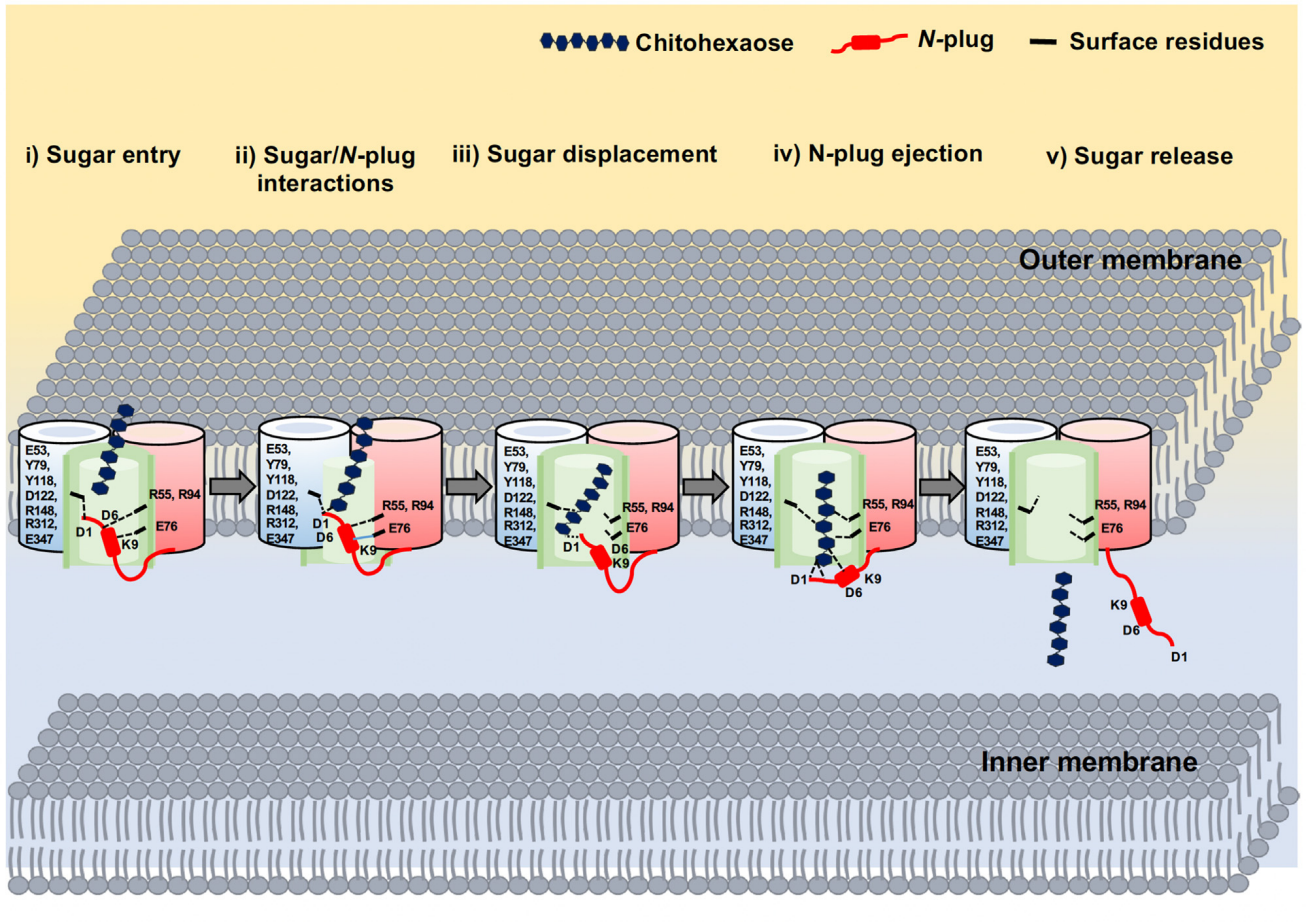
**The displacement model of sugar translocation through the *Vh*ChiP tunnel based on SMD simulation data.** Chitohexaose is shown as a string of hexagonal units (*blue*), the *N*-plug is shown in *red*, *dashed lines* represent hydrogen bonds, *dashed lines with arrows* indicate the direction of sugar and the *N*-plug movement. SMD, steered molecular dynamics.

The crystal structures suggested that deletion of the *N*-plug did not interfere with trimer formation by *Vh*ChiP because the three subunits were held together by intramolecular L2–L2 interactions (22). However, the most critical effects were observed in the crystal structures in complex with chitohexaose, where five key H-bonds that are formed with Arg^94^, Asp^135^, Asp^147^, Arg^148^, and Arg^312^ around the central affinity sites were missing in the truncated structure (See Fig. 3, residues highlighted in green), leading to a 22.3-fold reduction in the binding affinity for chitohexaose (Table 2). In our previous results, we also demonstrated by proteoliposome swelling assays that truncation of the channel caused a 2-fold reduction in the rate of permeation of bulk sugar molecules, as compared to the WT channel (20). The loss of the H-bond interactions in the structure of truncated *Vh*ChiP in complex with sugar and the drastic reduction in the binding affinity as demonstrated by single-channel electrophysiology and ITC binding studies suggested that the *N*-plug is displaced and subsequently ejected during sugar translocation. The initial interaction of the sugar chain would be through H-bonding with Asp^1^ at the tail of the *N*-plug, as is supported by the large reduction in the binding affinity produced by mutation of Asp^1^ to Ala. The structural details and electrophysiology data, supported by the SMD simulations, suggest that sugar translocation occurs in a multistep process, as shown in Figure 9. Step 1: Sugar entry. Chitohexaose enters the plugged pore and resides in the upper part of the protein lumen, while the *N*-plug occupies the bottom half of the pore. Step 2: Sugar/*N*-plug interaction. The incoming sugar moves downward, the reducing end of GlcNAc-1, making contact with Asp^1^ of the *N*-plug through a strong H-bond. The interaction between chitohexaose and the *N*-plug peptide was confirmed by ITC and site-directed mutation studies of Asp^1^. Step 3: Sugar displacement. The reducing end of the sugar protrudes further, constraining the *N*-plug and causing Asp^6^ and Lys^9^ to detach from the channel wall. Step 4: *N*-plug ejection. Protrusion of the sugar chain finally expels the *N*-plug from the pore on disruption of the hydrogen bonds between the sugar molecule and the tail of the *N*-plug. Step 5: Sugar release. The release of the sugar chain occurs almost simultaneously with *N*-plug ejection.

## Concluding remarks

In this study, we determined the crystal structures of truncated *Vh*ChiP, lacking the *N*-plug, in the absence and presence of chitohexaose. Structural analysis of sugar–channel interactions suggested that *N*-plug deletion led to the loss of five hydrogen bonds that interacted with the GlcNAc moieties at the central affinity site, thus drastically weakening the binding affinity of the truncated channel. SMD simulations suggested that the movement of the sugar molecule along the protein pore ejected the *N*-plug, while at the same time, hydrogen bonds formed between the reducing end of the sugar chain and the *N*-plug facilitate sugar translocation. Since several *Vibrio* species, including *V. campbellii*, are known to cause severe *Vibriosis* in marine organisms, such as fish, shrimps, and corals, understanding the mechanistic detail of sugar translocation may help the strategic design of powerful nutrient-based antimicrobial molecules and *N*-plug mimicking antimicrobial peptides that specially target ChiP channels, as potential therapeutics against *Vibrio* infections.

## Experimental procedures

### Gene synthesis, recombinant protein expression, and purification

The *chi*P gene fragment, encoding the truncated *Vh*ChiP lacking the *N*-terminal amino acids 1-19, was synthesized by GenScript, then cloned into pET23a(+) vector. The *chiP* gene was designed to exogenously express the truncated channel with an intrinsic 25-aa signal peptide for insertion into the OM of the Omp-deficient *E. coli* host strain BL21 (DE3) Omp8 Rosetta. The recombinant WT and truncated *Vh*ChiPs were expressed and purified, following the protocol reported previously (11). Briefly, transformed cells were grown at 37 °C in LB liquid medium containing 100 μg mL^−1^ ampicillin, 25 μg mL^−1^ kanamycin, and 1% (w/v) glucose. At an *A*_600_ of 0.6 to 0.8, *IPTG* was added to a final concentration of 0.5 mM. Cell growth was continued for a further 6 h, and cells were then harvested by centrifugation at 4500*g* at 4 °C for 20 min. The cell pellet was resuspended in a buffer containing 20 mM Tris–HCl, pH 8.0, 2.5 mM MgCl_2_, 0.1 mM CaCl_2_, 10 μg mL^−1^ DNase*I*, and 10 μg mL^−1^ RNase*A*. Cells were lysed by sonication on ice for 10 min using a Sonopuls Ultrasonic homogenizer with a 6-mm diameter probe. The recombinant *Vh*ChiP was extracted with 2% (w/v) SDS, followed by incubation at 50 °C for 1 h with gentle stirring and centrifugation at 40,000*g* at 4 °C for 60 min. *Vh*ChiP was extracted from the pellets, which were enriched in OMs, in two steps. In a pre-extraction step, the pellet was washed with 15 ml of 0.125% *n*-octylpolyoxyethylene in 20 mM phosphate buffer pH 7.4 (ALEXIS Biochemicals), homogenized with a Potter–Elvehjem homogenizer, incubated at 37 °C for 60 min, then centrifuged at 100,000*g*, 4 °C for 40 min. In the second step, the pellet from centrifugation at 100,000*g* was resuspended in 10 to 15 ml of 3% (v/v) *n*-octylpolyoxyethylene in 20 mM phosphate buffer pH 7.4, then homogenized with a Potter–Elvehjem homogenizer, and incubated at 37 °C for 60 min, followed by further centrifugation at 100,000*g*, 4 °C for 40 min. After exchange of the detergent with 0.2% (v/v) lauryldimethylamine oxide (LDAO, Sigma-Aldrich) by thorough dialysis, *Vh*ChiP was further purified by ion exchange chromatography using a HiTrap Q HP prepacked column (5 × 1 ml), connected to an ÄKTA Prime plus FPLC system (GE Healthcare Life Sciences, Life Sciences Instruments, ITS Co Ltd). Bound proteins were eluted with a linear gradient of 0 to 1 M KCl in 20 mM phosphate buffer, pH 7.4 containing 0.2% (v/v) LDAO. *Vh*ChiP-containing fractions were pooled and further purified by size-exclusion chromatography using a HiLoad 16/600 superdex 200 (GE Healthcare). For site-directed mutation of Asp1 to Ala (mutant D1A), the mutated gene was synthesized and cloned into pET23a(+) vector by GenScript. Gene expression and purification of the D1A mutant was carried out as described above. The purity of the purified proteins was confirmed by SDS-PAGE analysis. Fractions containing only *Vh*ChiP were pooled, and the protein concentration was determined using the Pierce bicinchoninic acid protein assay kit (Bio-Active Co, Ltd).

### Protein crystallization and X-ray data collection and processing

Single crystals of the truncated *Vh*ChiP were grown successfully under several conditions from MemChannel and MemTrans Screen kits (Molecular Dimensions Limited). Single crystals of the ligand-free truncated *Vh*ChiP appeared in a sitting drop screen plate within 3 days at 291 K in MemTrans Screen kit in condition A2, containing 0.1 M lithium sulfate, 0.1 M N-(2-acetamido)iminodiacetic acid pH 5.2, and 26% (*v/v*) PEG 400. This condition was chosen for further optimization by varying the pH and PEG 400 concentration of the precipitant. Larger single crystals with an average size of 100 × 200 μm^2^ (W × L) were finally obtained from microseeding optimization after 3 days of incubation at 298 K. Single crystals of the truncated *Vh*ChiP cocrystallized with chitohexaose were also successfully grown in MemChannel Screen kit in condition C7, containing 0.125 M lithium nitrate, 0.1 M glycine pH 9.8, and 45% v/v PEG 400. Single crystals appeared within 5 days at 291 K. This condition was further optimized by seeding, and the final crystal size was on average 100 × 300 μm^2^ (W × L). Diffraction data were collected on beamline TPS 05A at the NSRRC (100 *K* at 1 Å). Data were indexed, integrated, and scaled with HKL2000 (30, 31). The structure was determined using MR, and structural factors were anisotropy-corrected. WinCoot (32) was used for manual model building, and the structure was refined with PHENIX (https://hexdocs.pm/phoenix/Phoenix.Endpoint.html) (33).

### Single-molecule electrophysiology

Single-channel analysis using the BLM reconstitution technique was performed as described previously (13, 34, 35). A diagram summarizing the BLM setup and the experimental design is shown in Fig. S4. The lipid bilayer cuvette consisted of a 25-μm thick Teflon film sandwiched between two chambers. The film had an aperture of 50- to 100-μm diameter, across which a virtually solvent-free planar lipid bilayer was formed. The chambers were filled with electrolyte solution and Ag/AgCl electrodes immersed on either side of the Teflon film. The electrolyte used was 1 M KCl buffered with 20 mM Hepes, pH 7.4. 1,2-diphytanoyl-sn-glycero-3-phosphatidylcholine (Avanti Polar Lipids) was used for lipid bilayer formation. To form the bilayer, the aperture was first prepainted with 1 μl of 1% (*v/v*) hexadecane in pentane (Sigma-Aldrich). One of the electrodes was used as the ground (*cis*), while the other electrode (*trans*) was connected to the head-stage of an Axopatch 200B amplifier (Axon Instruments). *Vh*ChiP (20–100 ng μL^−1^) was added to the solution on the *cis* side of the lipid membrane. At applied transmembrane potentials of ±100 mV, a single channel was frequently inserted within a few minutes. The protein solution in the chamber was then gently diluted by multiple additions of the working electrolyte, to prevent multiple insertions. Single-channel current measurements were performed in the voltage-clamp mode, with the internal filter set at 10 kHz. Amplitude, probability, and single-channel analyses were performed using pClamp *v*.10.5 software (https://www.moleculardevices.com/products/axon-patch-clamp-system/acquisition-and-analysis-software/pclamp-software-suite) (all from Molecular Devices). To investigate sugar translocation, chitooligosaccharide was added to the *cis* side of the chamber at a final concentration of 5 μM. Occlusions of ion flow caused by sugar diffusion through the inserting channel were usually recorded for 2 min. To determine the kinetics of sugar translocation on individual subunit blockages, discrete concentrations of chitohexaose (0, 0.25, 0.5, and 1.0 μM) was tested. To study channel blocking behaviors of the *N*-plug, the first nine amino acids (Asp^1^-Lys^9^) (the so-called *N*-plug peptide) that plugged the channel was synthesized (GenScript). Two concentrations of the peptide (10 and 40 μM) were added to either the *cis* or the *trans* side of the chamber, containing with or without 1 μM of chitohexaose on the *cis* side.

The equilibrium binding constant *K* (M^-1^) was estimated from the decrease in the ion conductance in the presence of increasing concentrations of sugar using the following equation (36):

(1) $$G_{\max-}G_{c}/G_{\max}=I_{\max-}I_{c}/I_{\max}=K\left[C\right]/\left(K\left[C\right]+1\right)$$

*G*_max_ is the average conductance of the fully open *Vh*ChiP channel, and *G*_c_ is the average conductance at a given concentration [c] of chitohexaose. *I*_max_ is the initial current through the fully open channel in the absence of sugar, and *I*_c_ is the current at a particular sugar concentration. The titration experiments were also analyzed using double reciprocal plots.

### Binding study by ITC

ITC experiments were carried out at least three times using the MicroCal PEAQ-ITC (Malvern Instruments Ltd) at 25 ± 1 °C with a stirring speed of 500 rpm. For titration experiments, 40 μl of chitohexaose or the *N*-plug was titrated from a syringe into the 300 μl-calorimeter cells containing 0.05% (*v/v*) LDAO, 20 mM sodium phosphate buffer, pH 7.5, and protein solution. The optimized concentration ratios of chitohexaose and *N*-plug to *Vh*ChiP variants are shown in Table 3. Injections (2 μl per injection) were repeated 19 times over 150-s intervals. The background was measured by injecting the corresponding ligand into the cell containing only the buffer. The ITC data were collected and analyzed using the MicroCal PEAQ-ITC analysis software (https://www.malvernpanalytical.com/en/support/product-support/software/microcal-peaq-itc-analysis-software-v141). The ITC profile obtained by injecting the corresponding ligand into the reaction cell containing buffer without *Vh*ChiP was subtracted from the control dataset. The resultant data were fitted by a single-site binding model with the nonlinear least square algorithm.

### SMD simulations

The ligand-free, OM-expressed *Vh*ChiP (PDB ID: 5MDQ) was used as the starting structure since the *N*-plug resides inside the pore. Hydrogen atoms were added to *Vh*ChiP, while all water molecules, ions, detergents, and other ligands were removed from the crystal structure. Docking parameters were adjusted by default using the automatic genetic algorithm parameter setting in GOLD (37, 38, 39). All amino acid residues of *Vh*ChiP located within a 12 Å radius from the extracellular side were selected to define the sugar-binding sites. Iterated cycles of docking simulations were performed, generating 100 possible ligand conformers. All the GOLD docking calculations were further inspected for protein–ligand interactions. The conformer with the highest ChemPLP docking score of 77.5 was selected as the optimal protein-ligand model for MD simulations. The virtual biological system for MD simulations was set by mimicking *Vh*ChiP in the lipid membrane (40). The solvation system, containing chitohexaose-bound *Vh*ChiP, phospholipid, water, and ions, was prepared using CHARMM-GUI (41, 42). The protein–chitohexaose complex was embedded in a 1-palmitoyl-2-oleoyl-sn-glycero-3-phosphoethanolamine bilayer with TIP3P water and 1 M KCl. Energy minimization was performed using the steepest descent algorithm for 5000 steps, followed by a 5 ns equilibration in the constant-temperature, constant-pressure ensemble (NPT) with positional restraints on the heavy atoms of the proteins and lipids. MD simulations were performed with the GROMACS-2022.2 (https://manual.gromacs.org/2022/install-guide/index.html) (43) using the CHARMM36 all-atom force field (44). The particle-mesh Ewald approach was used to calculate long-range electrostatic interactions with a cut-off of 12 Å. Short-range Coulomb and Lennard Jones interactions were explicitly calculated up to the cut-off distance. The LINCS algorithm was applied to constrain the lengths of all bonds containing hydrogen atoms (45). The simulations were carried out in the NPT ensembles, which could be achieved by a semi-isotropic Parrinello–Rahman barostat (46) at 1 bar with a coupling constant of 5 ps and the Nosé–Hoover thermostat (47, 48) with a coupling constant of 1 ps. Energy minimization was carried out at 303 K to equilibrate the system, which included a sugar–protein complex embedded in phospholipids with water and KCl in the aqueous phase. SMD simulations were run under stabilized parameters, as shown in Fig. S5. The potential energy was initially decreased and became stable after 500 energy minimization steps (Fig. S5*A*). The solvation system containing phospholipid 1-palmitoyl-2-oleoyl-sn-glycero-3-phosphoethanolamine in the NPT ensembles became well-equilibrated within 5 ns of simulation time with an average temperature of 303.15 ± 0.125 K (Fig. S5*B*), the average pressure of 5 ± 50 bar (Fig. S5*C*), and the density of solvation of 1065 ± 2 kg m^−3^ (Fig. S5*D*). SMD simulations were subsequently performed to predict the synchronized movements of the chitohexaose chain and the *N*-plug using GROMACS tools. The sugar chain was pulled by the center of mass away from the active site vestibule. The pulling velocity of 0.01 nm^−1^ ps^−1^ and the bias force constant of 750 kJ.mol^−1^ nm^−2^ was applied in the unbinding and entry process. One-dimensional reaction coordinates were related to the *z*-coordinate distance between the center of mass of the sugar molecule. A series of configurations and reaction coordinates across the tubular pore were acquired, generating 650 configurations for every 0.5 Å of the sugar movement.

## Data availability

All the data used for this study are available upon request to wipa.s@vistec.ac.th.

## Supporting information

This article contains supporting information.

## Conflicts of interest

The authors declare that they have no conflicts of interest with the contents of this article.
