# Supplementary material for: Structural displacement model of chitooligosaccharide transport through chitoporin
Source: J Biol Chem. 2023 Jul 1;299(8):105000. doi: 10.1016/j.jbc.2023.105000 (PMC10406626; doi:10.1016/j.jbc.2023.105000)
Supplement: Supplemental Figures [file mmc2.docx]

**Supplemental figures**


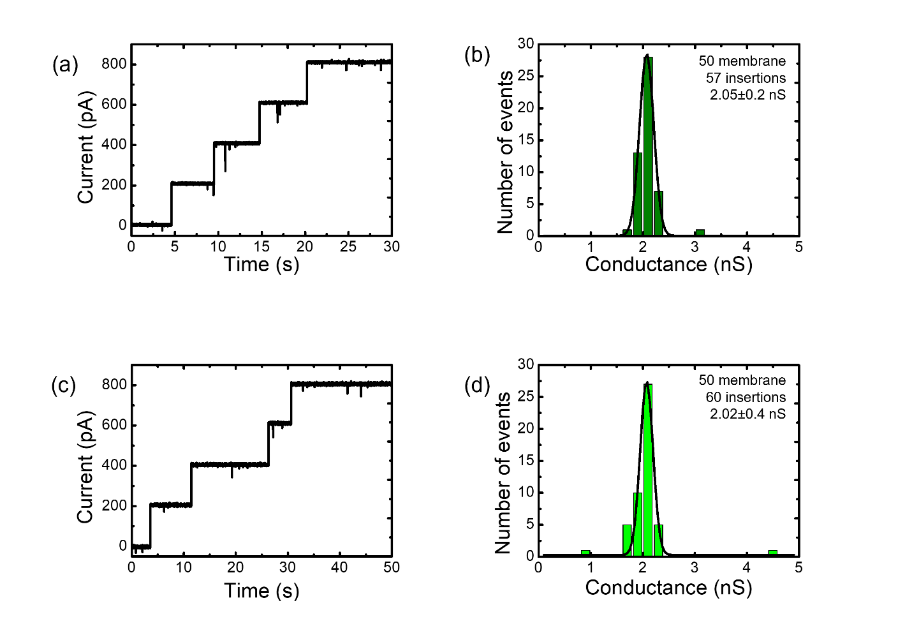


**Figure S1**. Ion current recordings obtained by the black lipid membrane (BLM) reconstitution technique. BLM measurements of successive insertions of *Vh*ChiP trimers at a transmembrane potential of +100 mV. Lipid bilayers were formed across a 200-µm aperture by the ‘painting’ technique using 50 mg.mL^-1^ azolectin in *n*-hexane and bathed on either side in 1 M KCl. *Vh*ChiP (1.0 mg.mL^-1^) was added on the *cis* side and ion current traces acquired for 50 s. Fast insertion of one *Vh*ChiP occurred within millisecond time-resolution, and current traces are shown for WT **(a)**, and truncated **(c)** *Vh*ChiP. The traces represent multiple insertions of *Vh*ChiP variants produced by an applied membrane potential of +100 mV. The histograms from the corresponding traces gave the probability of a pore conductance (*G*) averaged over several hundred inserting channels as indicated. The black line represents a single Gaussian fit shown as WT **(b)** and truncated **(d)** of *Vh*ChiP.


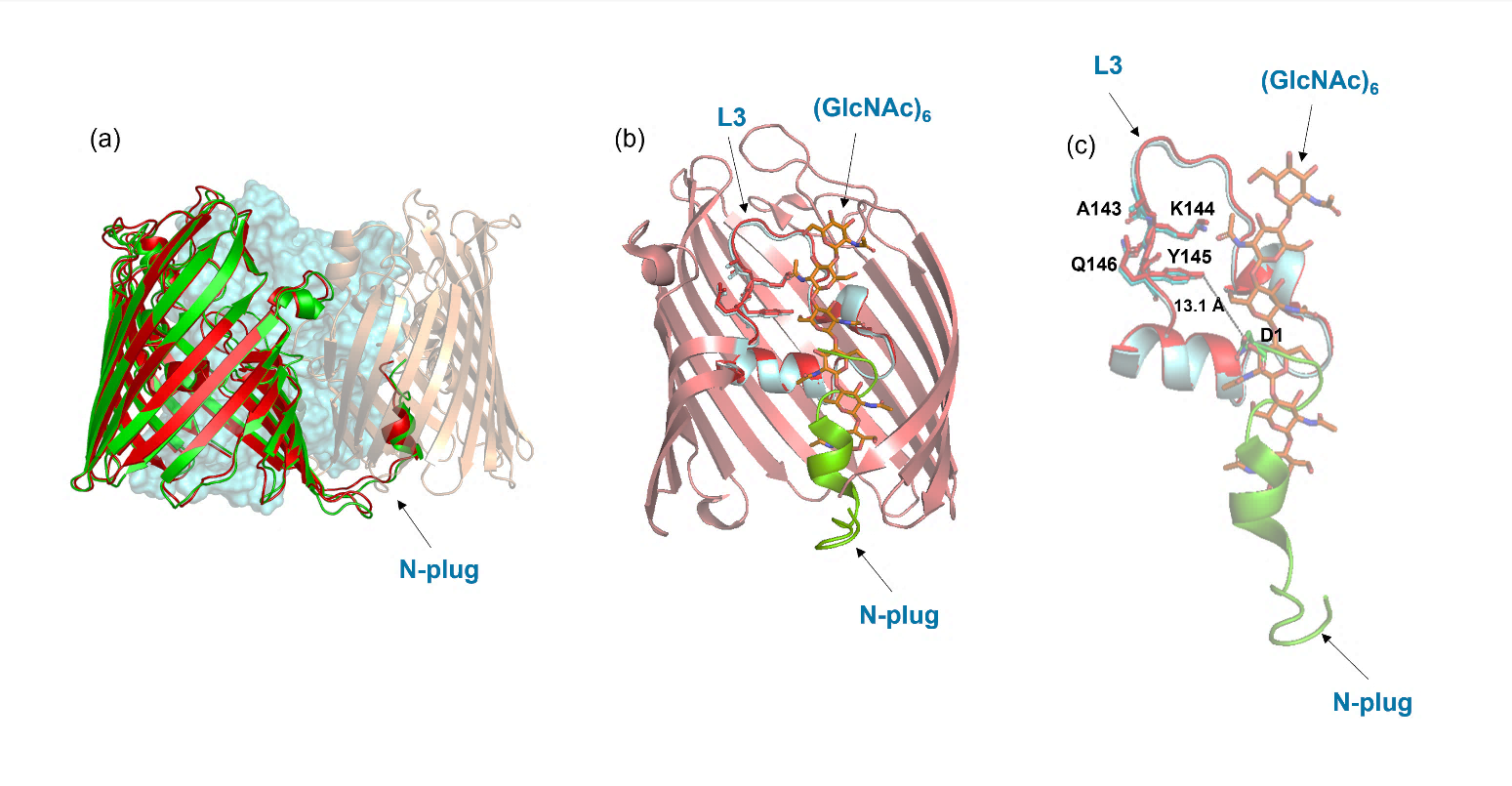


**Figure S2.** Stability of the *N*-plug and movement of loop L3 of *Vh*ChiP inside the pore in the absence and presence of chitohexaose. (a) Superimposition of the *N*-plug before (t = 0) and after (t = 50 ns) SMD simulations, (b) Movement of loop L3 inside the empty channel (PDB ID: 5MDQ) as compared to the sugar-bound channel (PDB ID: 5MDR), (c) Superimposition of the L3 residues Ala^13^ - Gln^146^ of the *Vh*ChiP channel with and without sugar.


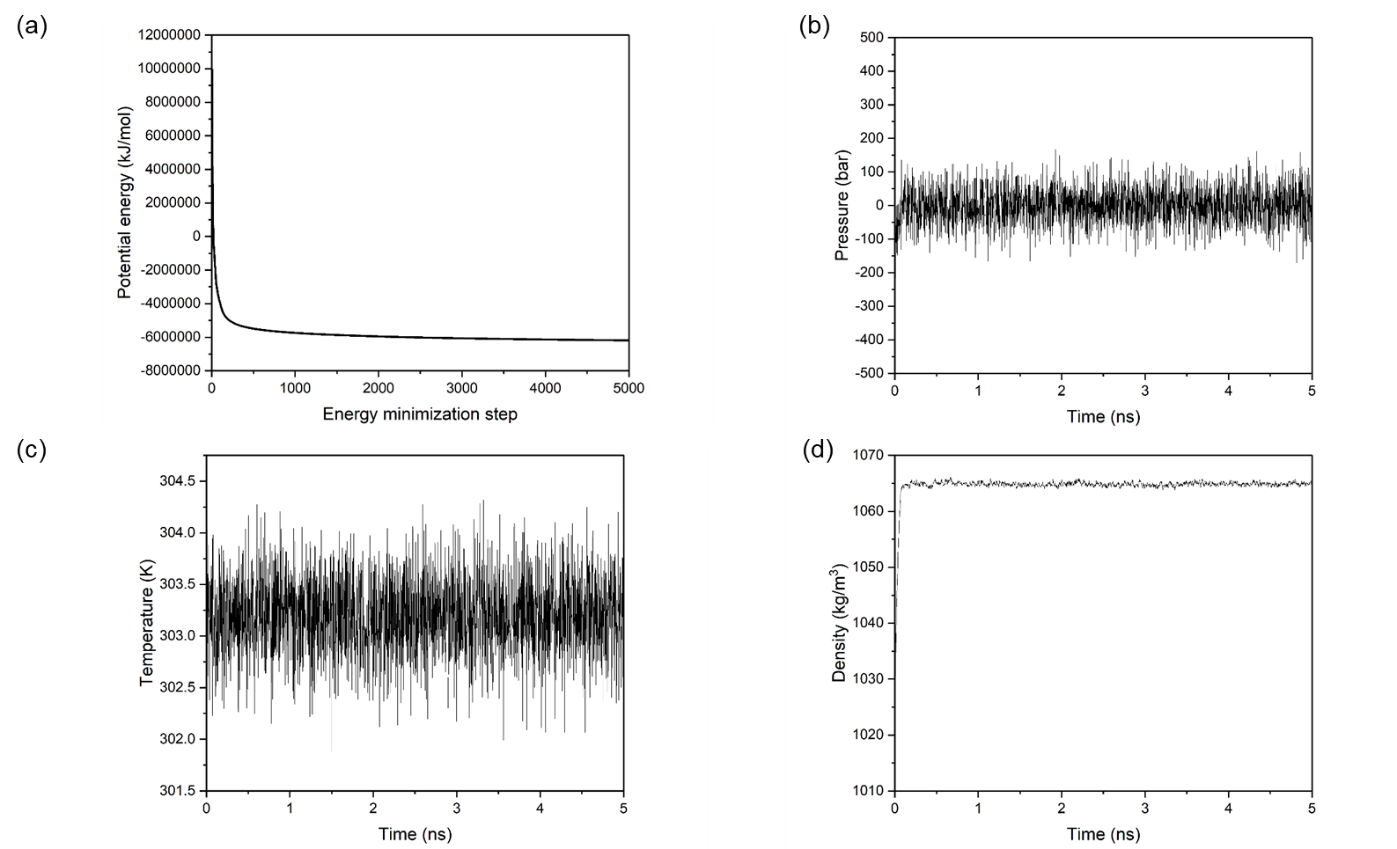


**Figure S3.** Parameter optimization for SMD. (a) The energy minimization using the steepest descent algorithm for 5000 steps. (b) The 5-ns equilibration system with a constant pressure of 5 ± 50 bar, (c) The 5-ns equilibration system with constant temperature of 303 kelvin, and (d) the stability of density of solvation observed over 5 ns.


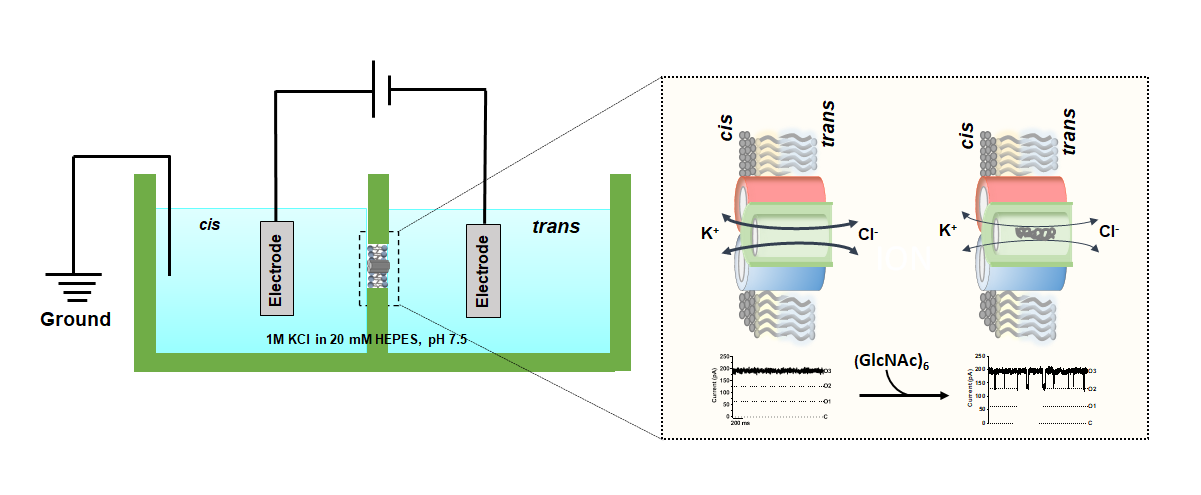


**Figure S4.** A diagram explaining the principles of the black lipid membrane reconstitution (BLM) technique that was used for single channel recordings.


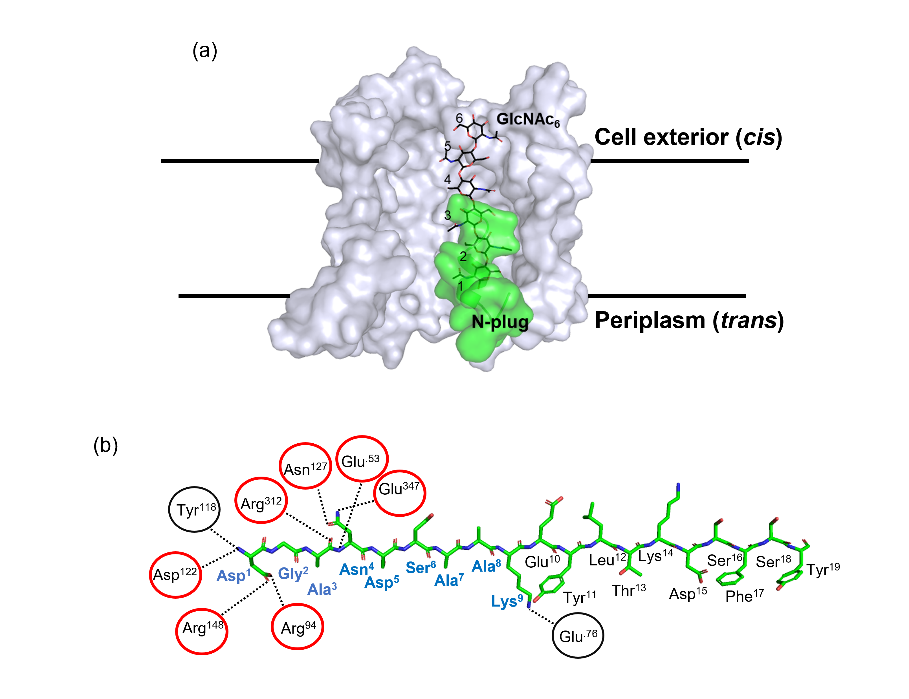


**Figure S5.** The pore-lining residues shared by the sugar ligand and the *N*-plug inside the protein channel. (a) Superimposition of *Vh*ChiP with chitohexaose (PDB id 5MDR) and the plugged channel (PDB id. 5MDQ) showing the overlapping of the *N*-plug and the sugar molecule in the bottom half of the pore (assigned as affinity sites 1 to 3). (b) The analysis of interactions between the *N*-terminal segment and active surface residues before sugar displacement, with red circles showing the amino acid residues that make overlapping interactions with the *N*-plug and the sugar molecule.
