# Supplementary material for: Structural displacement model of chitooligosaccharide transport through chitoporin
Source: J Biol Chem. 2023 Jul 1;299(8):105000. doi: 10.1016/j.jbc.2023.105000 (PMC10406626; doi:10.1016/j.jbc.2023.105000)
Supplement: Supplemental Tables [file mmc3.docx]

**Supplemental Table**

**Table S1.** Hydrogen bonds and hydrophobic interactions participating in substrate binding to the truncated and WT *Vh*ChiPs. The interactions were obtained from LIGPLOT (**ref. 36**) analysis as depicted in **Fig. 3**. Affinity site 1 is located towards the periplasmic side, while affinity site 6 is located towards the extracellular side.

|  | | | **PDB id: 7EQR**  **(Truncated, this study)** | **PDB id: 5MDR**  **(WT, ref. 35)** |
| --- | --- | --- | --- | --- |
|  |  |  | Donor…. acceptor for hydrogen bond (Å) or residues for hydrophobic interaction | Donor…. acceptor for hydrogen bond (Å) or residue for hydrophobic interaction |
| **Affinity site** | 1 | Hydrogen bond | Tyr^349^…. HO-C6 (3.50 Å) | - |
|  |  | Hydrophobic interaction | Trp^123^, Tyr^349^ | Trp^123^, Tyr^349^ |
|  | 2 | Hydrogen bond | Asn^127^…. *O*-acetamido (3.02 Å), Glu^347^…. *N*-acetamido (3.24 Å) | Asn^127^…. *O*-acetamido (2.73 Å), Arg^312^…. *N*-acetamido (2.95 Å), and Glu^347^…. *N*-acetamido (2.95 Å) |
|  |  | Hydrophobic interaction | Trp^123^ | Trp^123^ |
|  | 3 | Hydrogen bond | Asp^122^…. *N*-acetamido (3.00 Å) | Glu^53^…. HO-C6(2.95 Å), Arg^94^ …. *N*-acetamido (2.91 Å), Asp^122^…. *N*-acetamido (2.95 Å), Arg^148^….O-acetamido (2.96 Å), and Arg^312^…. HO-C6 (2.90 Å) |
|  |  | Hydrophobic interaction | - | - |
|  | 4 | Hydrogen bond | Glu^53^…. *N*-acetamido (3.17 Å) | Asp^135^… HO-C3 (2.96 Å), and Asp^147^… HO-C6 (2.95 Å) |
|  |  | Hydrophobic interaction | Trp^136^ | Trp^136^ |
|  | 5 | Hydrogen bond | - | - |
|  |  | Hydrophobic interaction | Phe^84^, Trp^136^ | Phe^84^, Trp^136^ |
|  | 6 | Hydrogen bond | Asn^336^…. HO-C3 (2.85 Å), *N*-acetamido (3.00 Å) | Asn^336^…. HO-C3 (2.90 Å) |
|  |  | Hydrophobic interaction | Phe^84^, Trp^331^ | Phe^84^, Trp^331^ |
